# Tetanus Vaccination in Agricultural Workers: A Retrospective Study on Seroprevalence over 10 Years

**DOI:** 10.3390/vaccines12121363

**Published:** 2024-12-02

**Authors:** Ermanno Vitale, Veronica Filetti, Giorgio Bertolazzi, Gabriele Giorgianni, Nektaria Zagorianakou, Andrea Marino, Massimiliano Esposito, Vincenzo Restivo, Serena Matera, Venerando Rapisarda, Luigi Cirrincione

**Affiliations:** 1 Department of Medicine and Surgery, University of Enna “Kore”, 94100 Enna, Italy; ermanno.vitale@unikore.it (E.V.); giorgio.bertolazzi@unikore.it (G.B.); massimiliano.esposito@unikore.it (M.E.); vincenzo.restivo@unikore.it (V.R.); 2 Department of Clinical and Experimental Medicine, University of Catania, 95124 Catania, Italy; andrea.marino@unict.it (A.M.); serena.matera@yahoo.it (S.M.); vrapisarda@unict.it (V.R.); 3 Department of Prevention of Catania, Vaccination Unit, 95124 Catania, Italy; gabriele.giorgianni@aspct.it; 4 Cytology, University of Ioannina, 45110 Ioannina, Greece; nektaria.zagorianakou@gmail.com; 5 Department of Health Promotion Sciences Maternal and Child Care, Internal Medicine and Medical Specialties ‘Giuseppe D’Alessandro’, University of Palermo, 90127 Palermo, Italy; luigicirrincione@gmail.com

**Keywords:** tetanus vaccination, occupational medicine, epidemiology, immunization status, agricultural workers

## Abstract

Background/Objectives: Tetanus is a serious, non-contagious infection caused by *Clostridium tetani*, which remains a global health threat despite the availability of an effective vaccine. The current state of immunization for agricultural workers in Italy reveals significant disparities, reflecting a non-homogeneous distribution of vaccination coverage across regions and subgroups. The aim of the study was to investigate the prevalence of tetanus antibodies in a cohort of agricultural workers in Eastern Sicily in order to evaluate possible public health strategies for improving vaccination coverage. Methods: This observational retrospective study assessed tetanus immunization coverage in agricultural workers in Eastern Sicily during the period from 2012–2022. Results: A total of 1143 workers participated, of which 71% (n = 871) had protective tetanus antitoxin levels. Of the 835 vaccinated workers, 9% were not immune, while 19% of those who were not vaccinated or did not recall their vaccination history were immune. Significant gaps in vaccination were noted, particularly among non-European workers, with only 23% vaccinated compared to 89% of European workers. Additionally, vaccination rates were higher in those born after 1963, when vaccination became mandatory. Conclusions: The results underscore the need for targeted vaccination strategies, especially for older and migrant workers, as well as the importance of workplace immunization programs led by occupational physicians. Improving vaccination coverage among agricultural workers is essential for preventing tetanus infections in high-risk agricultural populations.

## 1. Introduction

Tetanus is an acute, non-contagious infectious disease caused by the bacterium *Clostridium tetani* [1]. This bacterium is commonly found in soil, dust, and animal feces, and it can enter the human body through cuts, wounds, or punctures. The spores are particularly resilient, capable of surviving extreme environmental conditions for extended periods. They can easily contaminate wounds, especially in outdoor or agricultural settings where contact with soil and organic matter is frequent. This risk is particularly pronounced in rural and agricultural regions, where direct interaction with potentially contaminated soil and equipment occurs daily, creating a continuous exposure pathway. Once inside the body, tetanus bacteria release a potent neurotoxin called tetanospasmin, which interferes with nerve signals and leads to severe muscle spasms, stiffness, and, if untreated, death [1,2]. The neurotoxin tetanospasmin acts by targeting the central nervous system, specifically by inhibiting the release of neurotransmitters such as GABA and glycine, which are critical for muscle relaxation [1,2]. In Italy, an annual incidence of 1.0/1,000,000 was reported, with 80% of cases occurring in subjects aged >64 years. The mean annual number of reported deaths was 21, with older adults showing a markedly higher case-fatality ratio compared to younger populations [3]. 

Despite the availability of an effective vaccine, tetanus remains a significant health concern worldwide, particularly in populations with inadequate immunization coverage [4]. According to a previous study evaluating seroprevalence in Italy, 19.2% of people were not susceptible to tetanus. The percentage of individuals with protective antibody levels had a peak of 87.0% in the age group 15–24 years, then progressively declined with increasing age to 43.4% in the age groups 45–64 years [3]. This decline underscores the importance of regular booster doses, especially for older adults, to maintain immunity and reduce the burden of tetanus-related morbidity.

In Italy, tetanus vaccination has been mandatory for children starting at 2 years of age and for certain occupational groups, including agricultural workers, since the enactment of Law 292/1963 [5]. Furthermore, the Italian Vaccination Plan recommends having a booster dose every 10 years to be always immunized [6].

Furthermore, the abolition of military conscription for individuals born after 1985 represents a turning point in Italy’s tetanus immunization strategy. Military medical examinations provided a structured opportunity for administering booster doses to young adults, ensuring a high level of immunity within this demographic [7]. However, the discontinuation of this practice has left a gap in immunization programs, which must now rely on alternative mechanisms to reach these populations effectively.

Agricultural workers are uniquely vulnerable to tetanus due to a combination of environmental exposure and occupational hazards. Activities such as plowing, handling manure, and working with livestock frequently expose workers to soil and organic matter laden with *C. tetani* spores [8]. Additionally, the high prevalence of minor injuries, including cuts, abrasions, and punctures from tools or machinery, further amplifies the risk. The seasonal nature of agricultural work may also contribute to inconsistencies in accessing healthcare services, complicating efforts to maintain adequate immunization coverage in the population of workers. Therefore, ensuring immunization for these workers is crucial for preventing tetanus disease [9,10].

The current state of immunization for agricultural workers in Italy reveals significant disparities, reflecting a non-homogeneous distribution of vaccination coverage across regions and subgroups. While the presence of primary healthcare services, such as general practitioners, ensures basic access to care, cultural factors often play a decisive role in influencing attitudes toward vaccination. In some cases, there is a pervasive underestimation of the risks associated with tetanus, compounded by limited awareness of the need for regular booster doses, particularly among older workers, who may view vaccination as unnecessary after childhood immunization [8,10].

Despite mandatory tetanus vaccination requirements, there is evidence that a significant sample of these workers may not be adequately protected [10,11]. This issue is further exacerbated by a cultural tendency to prioritize immediate occupational demands over preventive healthcare measures, which may lead workers to delay or neglect vaccinations [8].

Previous studies have highlighted gaps in immunization coverage and pointed out the need for more effective vaccination campaigns and health education programs [9,10]. In Italy, efforts to improve vaccination coverage have included public health campaigns and school-based vaccination programs for specific groups. However, challenges remain, particularly in ensuring that all at-risk populations are vaccinated. Agricultural workers who work in remote and rural areas compose one such population that requires focused attention [9,10]. 

The need to focus on agricultural workers in Eastern Sicily arises from the distinct demographic and occupational characteristics of the region. This area is marked by a high prevalence of agricultural activities, employing a substantial workforce that is regularly exposed to environmental and occupational hazards, thereby increasing the risk of tetanus infection.

This study aims to investigate the prevalence of tetanus antibodies in a cohort of agricultural workers in Eastern Sicily in order to evaluate possible public health strategies for improving vaccination coverage. 

## 2. Materials and Methods

### 2.1. Study Design

This observational retrospective study was conducted over a 10-year period, from 2012 to 2022, focusing on agricultural workers in Eastern Sicily. The study was designed in compliance with the Italian legislative decree D.Lgs. 81/08, which mandates regular health surveillance for workers in high-risk professions [12]. This legislative framework ensures a structured approach to identifying occupational health risks, including those related to preventable diseases such as tetanus. The retrospective nature of the study allowed for the analysis of historical data to identify trends and gaps in vaccination coverage among this population.

Data collection occurred during routine health surveillance visits, which are conducted periodically as part of mandatory occupational health protocols. These visits provided an opportunity to gather comprehensive demographic and clinical information on the participants, including age, profession, general health conditions, and vaccination history.

### 2.2. Population and Laboratory Analysis

The study population consisted of agricultural workers actively employed in Eastern Sicily, representing a demographic highly exposed to environmental and occupational hazards. Serological analyses were performed on all participants to assess the presence of protective levels of tetanus antibodies. The Tetanus ELISA IgG test kit was used, with a titer of >0.1 IU/mL considered protective.

The samples were collected and centrifuged at 4000 rpm for 10 min separate the serum, following standard laboratory protocols. The Biolab ELISA kit was utilized for quantification, involving a multi-step procedure that included the incubation of test samples, compliance with regulation standards, and the placement of control samples on ELISA plates pre-coated with tetanus toxoid antigen. HRP-conjugated antibodies were then added, followed by a substrate reaction and absorbance measurement at 450 nm. Calibration curves generated from known standards enabled the interpolation of antibody concentrations.

Participants were classified as vaccinated against tetanus if they self-reported receiving a tetanus vaccination within the last 10 years. Direct verification of booster doses was not possible due to the absence of a centralized vaccination registry, highlighting a limitation in data reliability. Workers were further stratified based on year of birth, distinguishing those born before and after 1963, the year tetanus vaccination became mandatory, and those born before and after 1986, a period marked by changes in healthcare policies.

### 2.3. Statistical Analysis

The socio-demographic and clinical characteristics of all the recruited workers were summarized using frequencies and percentages. In order to evaluate the distribution of quantitative variables such as age, a skewness and kurtosis test was performed. The mean and standard deviation (SD) were chosen for the normally distributed variables, while the median and interquartile range (IQR) were used for the non-normally distributed variables.

The differences in quantitative variables normally and non-normally distributed among workers were evaluated, with a Chi^2^ test used for the qualitative variables.

ANOVA or *t*-tests, which are appropriate for continuous variables, were not applicable, as the key variables in this study were qualitative in nature. This approach ensured the most appropriate evaluation of the data.

A *p*-value of <0.05 was considered statistically significant. The statistical analyses were performed using SPSS Statistics 25.0. Descriptive statistics were used to summarize the demographic and serological data. 

## 3. Results

The study sample consisted of 1143 males with a mean age of 49.3 ± 7.9 years. Of these, 566 (49%) were agricultural workers and 577 (51%) were farmers and ranchers, with a working seniority of 19.3 ± 4.2 years. Only 22% (n = 262) of them declared the use of agrochemicals (Table 1).

No workers reported suffering from autoimmune diseases or using immunosuppressive drugs. Of all enrolled workers, 73% (n = 835) declared that they were vaccinated. Among workers who reported that they had never been vaccinated or did not remember having been vaccinated (NVOR), 19% (n = 57) were immune when tested. Tetanus antitoxin levels >0.1 IU/mL were detected in 71% (n = 871) of all workers. Among vaccinated subjects (n = 835), there were 75 (9%) cases who were not immune (Table 2). No difference was observed between the categories of “agricultural” and “agricultural and ranchers” among workers.

Among the examined subjects, 58% (n = 668) were born after the introduction of mandatory vaccination in 1963. Of these, 89% (n = 596) were vaccinated against tetanus, while only 47% (n = 221) of those born before 1963 had received the vaccine. Regarding immunity, no significant differences were observed between groups born before or after 1968. Additionally, individuals born after 1985 (n = 263) exhibited significantly higher vaccination coverage (85%) compared to those born before 1985 (68%, *p* ≤ 0.05). However, the prevalence of immunity was similar between the two groups.

An analysis of geographical origin revealed a significant disparity between non-European and European workers. Specifically, only 23% (n = 62) of non-European individuals were vaccinated, compared to 89% (n = 773) of Europeans (*p* < 0.005). Furthermore, the immunity level among non-Europeans was substantially lower (26% versus 85%, *p* < 0.005) (Table 3).

## 4. Discussion

Despite advances in medical science, tetanus remains a persistent public health challenge globally, particularly in regions with inadequate healthcare access and suboptimal vaccination coverage [13,14]. Globally, similar trends in vaccination gaps have been observed in other high-risk occupational groups, such as construction workers and manual laborers, especially in regions with fragmented healthcare systems. For instance, in rural areas of low- and middle-income countries, tetanus vaccination coverage remains critically low due to logistical and economic barriers. This underscores the universal relevance of targeted immunization strategies to address occupational and regional disparities [10]. In Italy, the incidence of tetanus has decreased significantly since the introduction of mandatory vaccination, but cases still occur, predominantly among older adults and individuals with incomplete vaccination histories [9,10]. The findings of this study highlight significant gaps in tetanus immunization among agricultural workers in Eastern Sicily. Despite mandatory vaccination laws, 29% (n = 326) of the workers did not have protective levels of tetanus antitoxin, underscoring the need for targeted interventions. The age-related decline in seropositivity aligns with previous studies indicating waning immunity with increasing age, despite the absence of verifiable data in this study regarding the timing of the last vaccine dose [15]. Similar trends have been reported in studies conducted in other regions of Italy, such as central Italy, indicating that the issue is not localized, but reflects a nationwide public health concern [10]. The progressive decline in immunity with age could also be influenced by comorbidities that impact immune responses, such as diabetes or chronic respiratory conditions, which are more prevalent in older populations. Additionally, behavioral factors, including reduced health-seeking behaviors among older workers, may exacerbate these gaps in booster vaccination adherence. It is also possible that the lower level of immunization in older workers, although not significant, although significant, is linked to the non-mandatory nature of the tetanus vaccine for those born before 1963, the year in which the tetanus vaccination was made mandatory starting from the second year of life, as well as for some categories of workers. Although the booster should be administered every 10 years [16,17], it is not uncommon to find immunity even after a long time from the last administration of the vaccine [18,19]. In this cohort of workers, a significant disparity in vaccination rates was observed between individuals born before and after 1963, the year when mandatory tetanus vaccination was introduced in Italy. Specifically, only 47% (n = 222) of those born before 1963 were vaccinated, compared to 89% (n = 596) of those born after 1963, with a statistically significant difference (*p* ≤ 0.05). This highlights the long-term impact of public health policies in promoting vaccine coverage among younger generations.

Furthermore, it was observed that vaccination rates among individuals born before 1986 were markedly lower (68%) compared to those born after 1986 (85%, *p* ≤ 0.05). The higher rates observed in the latter group can be attributed to improvements in the healthcare system; in fact, despite the abolition of military service, where tetanus vaccinations were administered, vaccination coverage in Italy has not decreased, as the tetanus vaccine is still mandatory for newborns in co-administration with the pertussis and diphtheria vaccines [20,21]. However, despite the mandatory tetanus vaccination for those born after 1968 and for agricultural workers (Italian Legislation 292/1963), vaccination coverage in Italy is still low [5]. This low vaccination coverage has had an impact on the incidence of *C. Tetani* infections. Indeed, the ECDC [22] showed that Italy reported 40% (n = 131) of all cases reported in EU/EEA (n = 329) between 2017 and 2021. For the years 2019−2021, Italy reported the highest number of probable cases (90%) while for the years 2017−2018, they reported the highest number of confirmed cases (95%). 

Other characteristics of low immunization were related to country of origin. Indeed, there was a higher number of unvaccinated subjects among workers of non-European origin. This data is in agreement with current epidemiological data, which show that vaccination coverage rates in the countries of origin were comparable to those found among non-European workers in our study [23,24,25,26]. The data suggest that different vaccination policies across countries of origin and barriers such as communication barriers, including language and cultural factors, also impact access to information and trust in the immunization policies among migrants arriving in Italy [27,28,29]. Cultural misconceptions and a lack of tailored educational materials in the native languages of migrant workers may further hinder vaccination efforts. Developing culturally sensitive health communication strategies and engaging community leaders could enhance trust and promote greater adherence to vaccination programs among non-European workers. Furthermore, the lack of tetanus immunization could be due to a low level of attention among employers and occupational physicians in respecting the mandate to vaccinate workers from non-European countries before they enter the workplace.

It is, therefore, important to adopt vaccination promotion policies and strategies. 

Addressing these gaps requires a multifaceted approach. Public health campaigns tailored to the needs of agricultural communities, mobile vaccination units to reach remote areas, and targeted health education programs aimed at both local and migrant workers could significantly improve immunization coverage. One strategy could be to make the vaccine free for employers in order to eliminate economic obstacles that very often lie behind low adherence to tetanus vaccination. In addition to making vaccines free, providing financial incentives for employers to implement workplace vaccination programs could prove beneficial. Subsidies or tax credits for companies that ensure the complete vaccination of their workforce may address economic constraints while fostering a culture of health and safety. It is known that the implementation of strategies to increase access to vaccination services has led to an increase in adherence to DTP vaccination requirements [14]. It is also important for occupational physicians to implement vaccination activities in the workplace. As demonstrated by previous studies, the implementation of models that avoid contact with various pathogens has proven effective [30,31,32], but regarding transmissible diseases mediated by inanimate vectors, as in this case, it is necessary to pay attention to primary prevention through immunoprophylaxis [33]. Italian Legislation Decree 81/08 highlights how employers, after consulting an occupational physician, should adopt special protective measures for those workers for whom special protective measures are required, including personal health reasons and the provision of effective vaccines for those workers who are not already immune to the biological agents present in the work process [12]. The low prevalence of vaccination among non-European subjects requires greater attention from occupational physicians and from inspection bodies in order to guarantee adequate vaccination coverage for all workers exposed to the risk of tetanus. Indeed, gaps in vaccination coverage among agricultural workers pose not only immediate health risks but also significant long-term health and economic consequences. From a health perspective, low immunization rates can lead to an increase in tetanus cases, a severe disease requiring prolonged hospitalization and intensive care. This not only negatively impacts patients’ quality of life but also places an additional burden on healthcare systems, particularly in regions with limited resources, such as Eastern Sicily [34]. Also, the public health impact extends beyond tetanus alone. Non-adherence to mandatory vaccinations may signal a broader lack of trust in public health campaigns, negatively affecting other prevention initiatives. Addressing these vaccination gaps is therefore critical not only to mitigate the specific risks of tetanus, but also to strengthen the healthcare system as a whole, ensuring greater resilience against other public health challenges. A failure to address these systemic gaps could compromise the overall efficacy of Italy’s public health infrastructure. Strengthening the collaboration between occupational health services and public health authorities is crucial for creating a resilient framework capable of managing both routine immunizations and emerging health threats.

The main limitation of the study is related to the convenience sample recruited from a registry of all agricultural workers. Moreover, the lack of information regarding the tetanus vaccination history of workers is due to the unavailability of a worker vaccination registry. All the workers in the study were male, and sex-related differences in vaccination rates were therefore not applicable to this cohort.

Notwithstanding its limitations, this study provides an overview of the immunization status of a large cohort of agricultural workers evaluated over a 10-year time window. One of its strengths lies in the extensive temporal coverage, which enables a robust analysis of immunization trends and persistent gaps in a high-risk but underrepresented population. The focus on non-European migrant workers highlights specific barriers, such as cultural and communication challenges, emphasizing the need for tailored interventions. 

These findings offer a solid foundation for targeted vaccination campaigns and workplace health strategies, addressing systemic public health challenges effectively. Addressing these barriers will require a multifaceted approach, including the development of culturally sensitive educational materials and the involvement of community leaders to build trust and promote vaccine acceptance [35,36]. 

Future studies should aim to include a more diverse sample, including female workers and individuals from varied agricultural roles, to assess potential sex- and job-related differences in vaccination rates. Additionally, establishing a centralized vaccination registry would enable more accurate tracking of immunization histories and facilitate targeted interventions.

## 5. Conclusions

Vaccination is an important prevention tool that must be further adopted in the workplace. Agricultural workers are both more exposed to tetanus and at a higher risk of being inadequately immunized than other workers. Italy is still one of the European countries with the highest number of cases of tetanus infection. The data from this study show that agricultural workers in Eastern Sicily are not adequately immunized against tetanus. Of particular note is the poor coverage of non-European workers. These data points highlight the importance of finding targeted vaccination strategies to improve immunization rates, especially among vulnerable groups such as non-European workers. It will be important to adopt vaccination promotion strategies from healthcare authorities. Improving awareness about vaccination among employers and workers, along with removing potential economic and logistical barriers, can significantly enhance adherence to immunization programs. In the long term, fostering stronger partnerships between public health authorities and occupational health services will be essential. Such collaboration can create sustainable frameworks for routine immunization, ensuring that even vulnerable groups, such as migrants and seasonal workers, are adequately protected. Also, occupational physicians play a crucial role in adopting vaccination strategies for all workers exposed to the risk of contagion.

## Figures and Tables

**Table 1:** Enrolled workers characteristics.

Participants: n = 1143 Agricultural Workers	Mean ± SD or No. (%)
Age	49.3 ± 7.9
Agricultural Farmers and Ranchers	566 (49%)
	577 (51%)
Working Seniority (years)	19.3 ± 4.2
Smokers	354 (31%)

**Table 2:** Distribution of immune and non-immune individuals among vaccinated and non-vaccinated (NVONR) groups.

Immunization Status	Vaccinated (n = 835)	NVONR (n = 308)	*p*-Values
Immune (n = 817)	760 (91%)	57 (19%)	≤0.05
Non-immune (n = 326)	75 (9%)	251 (81%)	

**Table 3:** Immunization status based on year of birth and geographic origin.

	Vaccinated (n = 835)	NVONR (n = 308)	*p*-Values	Immune (n = 817)	Non-Immune (n = 326)	*p*-Values
Born After 1963 (n = 668)	596 (89%)	72 (11%)	≤0.05	468 (70%)	200 (30%)	n.s.
Born Before 1963 (n = 475)	221 (47%)	254 (53%)		349 (73%)	126 (27%)	
Born After 1986 (n = 263)	224 (85%)	39 (15%)	≤0.05	186 (71%)	75 (29%)	n.s.
Born Before 1986 (n = 880)	593 (68%)	287 (32%)		629 (71%)	251 (29%)	
Non-Europeans (n = 271)	62 (23%)	209 (77%)	≤0.05	73 (26%)	198 (74%)	≤0.05
Europeans (n = 872)	773 (89%)	99 (11%)		744 (85%)	128 (15%)	

## Data Availability

The original contributions presented in this study are included in the article. Further inquiries can be directed to the corresponding author(s).
